# Using tagging data and aerial surveys to incorporate availability bias in the abundance estimation of blue sharks (*Prionace glauca*)

**DOI:** 10.1371/journal.pone.0203122

**Published:** 2018-09-11

**Authors:** Milaja Nykänen, Mark Jessopp, Thomas K. Doyle, Luke A. Harman, Ana Cañadas, Patricia Breen, William Hunt, Mick Mackey, Oliver Ó Cadhla, David Reid, Emer Rogan

**Affiliations:** 1 School of Biological, Earth and Environmental Sciences, University College Cork, Cork, Ireland; 2 Aquaculture & Fisheries Development Centre, University College Cork, Cork, Ireland; 3 MaREI Centre, Environmental Research Institute, University College Cork, Cork, Ireland; 4 ALNILAM Research and Conservation Ltd, Madrid, Spain; 5 School of Geography, National University of Ireland, Galway, Ireland; 6 National Parks and Wildlife Service, Department of Culture, Heritage and the Gaeltacht, Galway, Ireland; 7 Marine Institute, Oranmore, Galway, Ireland; Department of Agriculture and Water Resources, AUSTRALIA

## Abstract

There is worldwide concern about the status of elasmobranchs, primarily as a result of overfishing and bycatch with subsequent ecosystem effects following the removal of top predators. Whilst abundant and wide-ranging, blue sharks (*Prionace glauca*) are the most heavily exploited shark species having suffered marked declines over the past decades, and there is a call for robust abundance estimates. In this study, we utilized depth data collected from two blue sharks using pop-up satellite archival tags, and modelled the proportion of time the sharks were swimming in the top 1-meter layer and could therefore be detected by observers conducting aerial surveys. The availability models indicated that the tagged sharks preferred surface waters whilst swimming over the continental shelf and during daytime, with a model-predicted average proportion of time spent at the surface of 0.633 (SD = 0.094) for on-shelf, and 0.136 (SD = 0.075) for off-shelf. These predicted values were then used to account for availability bias in abundance estimates for the species over a large area in the Northeast Atlantic, derived through distance sampling using aerial survey data collected in 2015 and 2016 and modelled with density surface models. Further, we compared abundance estimates corrected with model-predicted availability to uncorrected estimates and to estimates that incorporated the average time the sharks were available for detection. The mean abundance (number of individuals) corrected with modelled availability was 15,320 (CV = 0.28) in 2015 and 11,001 (CV = 0.27) in 2016. Depending on the year, these estimates were ~7 times higher compared to estimates without the bias correction, and ~3 times higher compared to the abundances corrected with average availability. When the survey area contains habitat heterogeneity that may affect surfacing patterns of animals, modelling animals’ availability provides a robust alternative to correcting for availability bias and highlights the need for caution when applying “average” correction factors.

## Introduction

Blue sharks (*Prionace glauca*) are considered to be one of the most abundant pelagic shark species, with one of the most extensive distributions of all elasmobranchs, ranging from 60°N to 50°S in tropical and temperate waters [1]. Data from tagging studies suggest that blue sharks are highly migratory, with trips of up to tens of thousands of kilometres [2,3], and show evidence of sex and size segregation [3,4]. Blue sharks are also the most heavily exploited shark species, being caught mainly as bycatch in the longline and drift-net fisheries for tuna and swordfish [5,6], but they are also targeted by commercial and recreational fisheries [7,8]. Like all large elasmobranchs, blue sharks are especially vulnerable to overfishing and bycatch due to their comparatively low reproductive rates and therefore decreased potential for population recovery [9]. Data on the stock status of the Atlantic population indicate severe and rapid regional declines [7,10] as well as a large overall decline in the Northwest Atlantic [11], supporting the listing of blue shark as ‘Threatened’ by the International Union for Conservation of Nature (IUCN) Red List. However, as data from other areas of its range are lacking or have high levels of uncertainty, the species is currently listed overall as ‘Near Threatened’ [12].

Stock assessments for sharks are typically done as a relative index of abundance, or catch-per-unit-effort (CPUE;, e.g. [13–15]), using landings or bycatch data. This approach can be problematic as most bycatch remains poorly reported [16–18]. In addition, further difficulties arise due to differential selectivity of the fishing gear [19]. However, recent efforts have been made to derive more reliable estimates of abundance for a number of shark species using non-fishery related methods including photo-identification [20], and aerial survey abundance estimation of whale sharks (*Rhincodon typus* [20]), basking sharks (*Cetorhinus maximus* [21]), lemon sharks (*Negaprion brevirostris* [22]) and giant devil rays (*Mobula mobular* [23]).

As with all shark species, blue sharks spend time at depth (e.g. [24,25]), with patterns of vertical movements varying with time of day [26], reproductive status [3], location in stratified off-shelf or well-mixed coastal waters [2], or between individuals [2,25]. The variation in diel depth preferences within the water column and the fact that blue sharks may only spend a portion of their time at the surface makes them unavailable for detection by observers from aerial survey platforms for an unknown proportion of the survey effort. If not accounted for in abundance estimation, this availability bias may lead to severe underestimation of their numbers. To our knowledge, only two previous studies on elasmobranchs have accounted for the bias resulting from the observation process (perception bias), as well as for availability bias [21,23]. Here we derive the first unbiased abundance estimate for blue sharks in a large area of the Northeast Atlantic by combining aerial survey data and distance sampling methodology [27,28] with models of availability for detection from tagging data. This is the first attempt to account for spatial and temporal variability in surfacing behaviour of sharks to derive an unbiased large-scale abundance estimate for blue sharks that could be used in regional stock assessment.

## Materials and methods

### Survey design and abundance data collection

**Fig 1:**
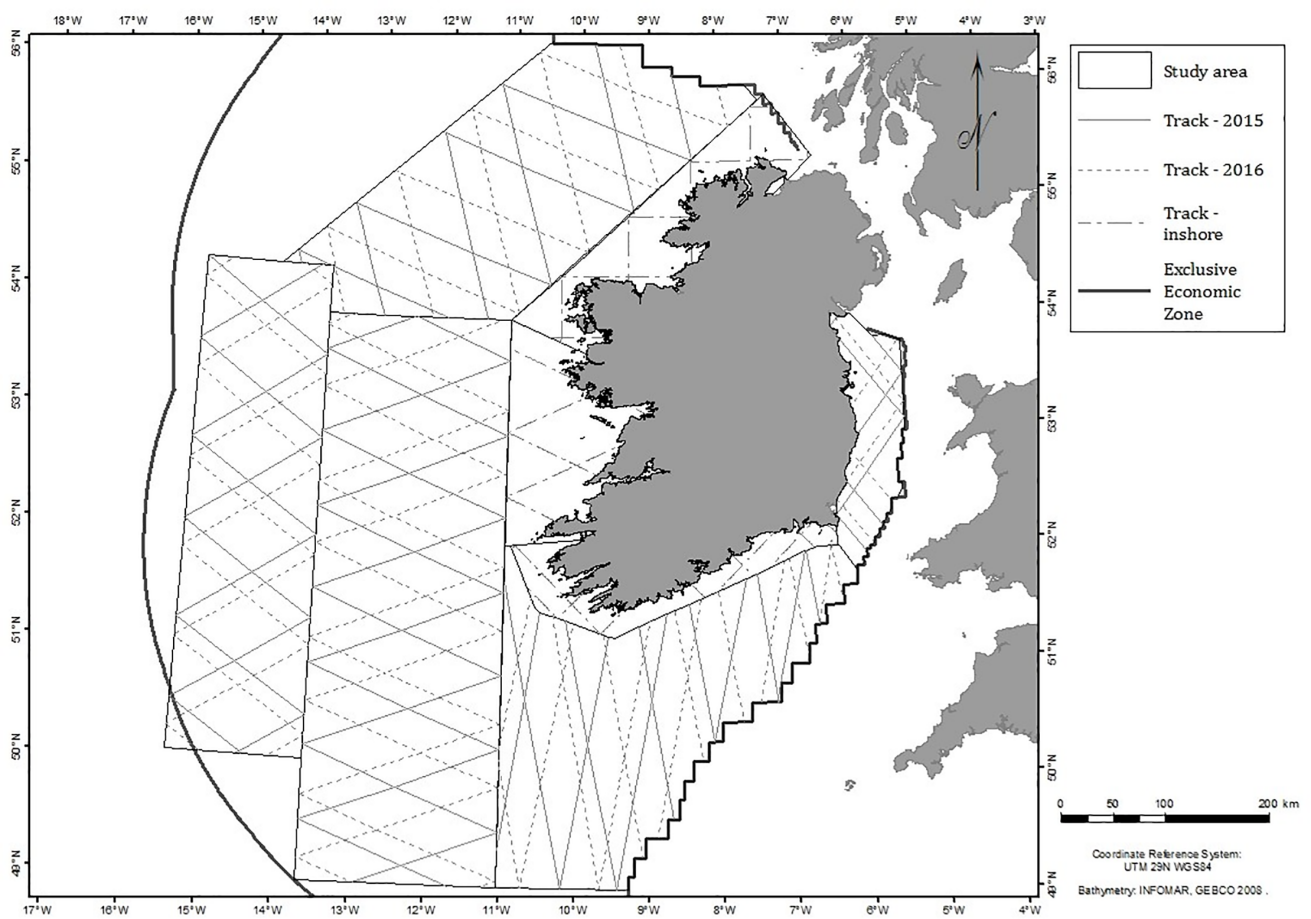
Aerial survey transects flown in the Irish Exclusive Economic Zone in 2015 (solid thin line) and 2016 (dashed lines). Note the added inshore tracks flown in 2016 only.

The data for the abundance estimation were collected during aerial surveys from a Britten-Norman BN-2 Islander fixed-wing aircraft equipped with bubble windows, with two observers located on either side of the plane. The plane was flown at an over ground speed of 90 knots (167 km/h) and an altitude of 183 m. Transect lines were designed to provide equal coverage probability for the survey area, and consisted of equally spaced randomly placed zig-zag lines (Fig 1). The transects were positioned differently for each year of survey, 2015 and 2016, to allow for more representative coverage of the study area and to avoid assumptions around animal distribution. The tracks were surveyed twice per year, once in the summer (May–July) and once in the winter (November–March), in both years (S1 Table).

During the aerial surveys, the plane’s geographic position was recorded every two seconds using an onboard GPS linked to a data logging computer. Observers recorded information on the Beaufort sea state, glare extent and severity, cloud cover, “subjective” sighting conditions (classified by the observers on each side as “good”, “moderate” or “poor”), water turbidity and surface reflectance at the beginning of each line and upon any change in the survey conditions. The observers searched an area extending out to 500 m from the plane’s trackline, and, upon detection of a shark, the perpendicular distance to the sighted animal was measured using clinometers when the animal was abeam of the aircraft. Sighting time, observation cue, behaviour of the animal, species identity and group size were also recorded.

### Environmental data acquisition

A spatial grid of resolution 0.10 x 0.06 decimal degrees (latitude x longitude) was created to cover the survey area, and physical and environmental variables were retrieved from different sources (see S2 Table) using the centre point of each grid cell and, for dynamic variables, for each season separately. Variables included water depth (m), slope index, and distances to shore and to the 200 m depth contour (latter as a proxy for distance to the continental shelf edge). Sea surface temperature (SST, C°), mixed layer depth (m) and chlorophyll-a concentration (mg m^-3^) were included as indices of marine hydrology and primary productivity. These variables were included in the abundance models to investigate their effect on the derived abundance of blue sharks following Cañadas and Hammond [29] and to predict the abundance over the survey area. As the tagging was done at a different time to the aerial surveys, the monthly means (with standard deviation) of SST and chlorophyll-a concentration across the entire area where the aerial surveys were conducted were plotted in order to investigate whether different environmental conditions prevailed during the tagging and the aerial surveys (see S1 Fig). The SST and chlorophyll-a data were retrieved as monthly composites from NASA website (https://oceancolor.gsfc.nasa.gov/cgi/l3) and the summary statistics were extracted in R.

### Blue shark depth data collection

All tagging procedures were approved by the Animal Experimentation Ethics Committee of University College Cork, and the blue sharks used in this study were tagged under licenses AE191130/I007 and AE19130/P002 issued by the Irish Health Products Regulatory Authority, compliant with the EU Directive 2010/63/EU for scientific research on animals.

The blue sharks were captured on rod and line by expert anglers using barbless non-offset circle hooks which minimises hook damage to the shark and highly reduces the chance of deep hooking. The hooks were “set” as soon as the sharks took the bait (mackerel) and were landed as quickly as possible to minimise the chance of exhaustion from the struggle. Stress to the animal once on deck was minimised by placing a flow of sea water into the shark’s mouth to ensure a continuous supply of oxygen to the gills for the duration of the procedure, which lasted no longer than 15 minutes.

Two blue sharks used in this study, a sub-adult female (shark A) and a juvenile of unknown sex (shark B), were fitted with popup satellite archival tags, or PSATs (MK10, Wildlife Computers, Redmond, WA, USA), in September 2010 and September 2012, respectively, approximately 20 km south of Cork, Co. Cork, Ireland. The tags were attached by placing a 20 cm long rubber sleeved monofilament tether (250 lb. test) through a single perforation made with a stainless steel drill (4 mm diameter) and were programmed to release after 120 days (shark A) and 70 days (shark B). The tags are positively buoyant, and trailed behind the swimming sharks during the deployment. PSATs are used to track large-scale movements and behaviour of marine animals whose surfacing times are too short to allow real-time transmission of data to the ARGOS-satellite system. Instead, data on depth, temperature and light intensity are collected during the deployment and sent to the satellite on release. PSATs were configured to record data in depth bins in 6-hour intervals (i.e., the data were recorded as the proportion of time spent during the six hours in different depth bins, for example, at depths of 0–1 m, 1–5 m, or 5-10m, and so on). Light intensity data was available for shark B only; the most likely swimming path was reconstructed for this shark by calculating geolocation trajectories (see S2 Fig).

### Modelling time at the surface

Data collected over the first two days after the tag deployment were removed to exclude any abnormal behaviour caused by the stress from tagging. We modelled the availability for detection as the proportion of time that tagged sharks spent in surface waters (0–1 m depth) during each six-hour time bin, and examined whether the time of day (morning or afternoon) or the sharks’ location (on-shelf versus off-shelf) had an impact on time at the surface. Since PSATs do not directly record geographic position and no light intensity data was available for shark A, it was assumed that this shark was no longer swimming in continental shelf waters once its diving behaviour changed and it started to make dives to depths greater than 150–200 m. However, the reconstructed track for shark B showed that this shark stayed within the continental waters throughout the tagging period (S2 Fig). Time was entered in the model as a factor consisting of just two of the four six-hour bins (06:00–12:00 and 12:00–18:00) because the aerial survey abundance data were collected during daylight hours. Since the response variable (availability) was proportional and included some zero and one values (0 or 100% time spent at the surface), we chose to model availability with a zero-one inflated beta distribution using R-package ‘zoib’ [30]. This package uses Bayesian inference with Markov Chain Monte Carlo (MCMC) sampling to simultaneously estimate the linear predictor (average time spent at the surface), and the probability of event of 1 (100% time at the surface) and 0 (0% time at the surface) [30]. We included shark ID as a random variable in the model, and accounted for temporal autocorrelation by dividing the data into daily blocks; therefore, the random variable in the models was an interaction term shark ID*Day. After testing for multi-collinearity between the covariates by calculating Generalized Variation Inflation Factors (GVIFs) [31] the candidate models were run with 50,000 MCMC iterations and 5,000 burn-in, with three independent chains per model. Convergence of the chains was confirmed by inspecting trace plots and potential scale reduction factor values. We compared the candidate models (with different factor combinations) using Deviance Information Criterion (DIC) values [32], and predictions of availability across grid cells covering the entire survey area were made using the best model. Predictions accounted for whether cells occurred either on or off the continental shelf (defined by average depth of under or over 150 m, respectively). Time of day was not included in predictive models as this could not be extrapolated beyond surveyed grid cells.

In addition, the R-package ‘GAMLSS’ [33] was used to model and visualize the proportion of time spent in the surface layer (0–1 m) by individual sharks. Similar to the zoib-model, this method handles proportional data (beta distribution) with zero and one values, but applies maximum likelihood instead of using Bayesian inference. The proportion of time spent at the surface was modelled as a function of time (observation), position (on-shelf or off-shelf) and time-bin (00:00–06:00, 06:00–12:00, 12:00–18:00 or 18:00–24:00). This approach was used mainly for its visualization possibilities that were unavailable in the zoib-package, and not for the predictions of availability due to the GAMLSS models including the random factor shark ID*Day failing to converge after several attempts.

### Abundance estimation

Abundance of blue sharks in the survey area was estimated separately for the summers of 2015 and 2016 using generalized additive models (GAMs) [34] and applying distance sampling methodology [28]. Firstly, bias in the observation process was quantified by estimating a detection function in R-package ‘Distance’ [35] based on the distances to the sighted animals, as well as including the potential effect of different factor variables on the detection probability: sea state, glare intensity, subjective conditions, turbidity and cloud cover. In order to fulfil the minimum observations required for fitting a detection function (*n* = 60 [27]), the detection function was derived using the detections of blue sharks and ‘unidentified sharks’ but not including basking sharks due to their significantly larger size, and therefore better detectability, compared to blue sharks. These observations of ‘unidentified sharks’ were likely to be blue sharks but in order to avoid inflating the abundance estimates, they were only used to estimate the detection function and were then excluded from the abundance models. Detection functions of different term compositions were compared with Akaike’s Information Criterion (AIC) values after confirming goodness-of-fit from quantile-quantile plots and Cramér–von Mises test statistic [36,37].

Once the best detection function was determined, density surface models (GAMs) were run using R-package ‘dsm’ [38] to predict the abundance of blue sharks for each grid cell of the survey area. We tested the effect of a combination of sighting location (interaction of x and y coordinates) and different covariates (depth, slope index, distance to shore, distance to the 200 m depth contour, chlorophyll-a, SST and mixed layer depth) on the observed count of blue sharks using a Tweedie error distribution with a log-link due to overdispersion in the data. Instead of correcting for availability after running the GAM, which often leads to larger overall variation in the final abundance estimate when an additional source of variation is added on top of the variation in the detection and count processes ([21,39]), availability for detection was entered directly into the density surface GAM by dividing the number of animals in an effort segment by their availability [38]. The estimated counts were thus scaled up if availability was <1. We modelled the abundance of blue sharks with three different measures of availability bias, 1) setting availability to 1, i.e. not applying any correction for availability; 2) setting availability to 0.41, the average time spent at the surface by both sharks during the tag deployment; and 3) setting availability as a vector of predicted mean availabilities for each grid cell depending on its location “on-shelf” or “off-shelf” based on the Bayesian zoib-model. The models were run with ‘REML’ as the fitting engine and leaving the gamma parameter at its default value (γ = 1.4) in order to avoid overfitting. The best fitting model was determined using shrinkage, where terms are selected out from the model as they are penalized to the zero function [40]. The fit of the models was confirmed by inspecting residual Q-Q plots, heteroscedasticity was checked by plotting the spread of the residuals to the linear predictors, and autocorrelation function plots were inspected for any autocorrelation in the data. Abundance for the study area was predicted with all three models including different measures of availability.

## Results

### Survey effort

Altogether 16,797 km of survey effort was flown during the summer and winter surveys in 2015–2016 (S1 Table). In the second year, 2016–2017, the survey effort totalled 20,387 km due to surveying an additional inshore area off the Irish coast (Fig 1). Twenty blue sharks were sighted in the summer 2015, and 35 were observed in the summer of 2016. No blue sharks were sighted during the winter surveys; therefore, their abundance was estimated only for the summers 2015 and 2016.

### Time at the surface

GVIFs of <1.5 indicated no collinearity between the co-variates in the availability models. The model including a random term consisting of shark ID and day, along with the fixed co-variates ‘shelf’ and ‘time bin’, was selected as the best model based on the negative difference in the DIC values (-10.76) when compared to a model including only the random term and the fixed factor ‘shelf’. Plotting the observed response to the model-predicted mean values indicated a reasonable fit of this model (S3 Fig). Both factors ‘shelf’ and ‘time bin’ had a significant effect on the linear predictor (mean proportion of time spent by sharks at the surface); time at the surface increased whilst on shelf and reduced during the hours of 12:00–18:00 (Table 1). The probability of sharks spending 100% of the time at the surface increased during the afternoon (Table 1). The predicted mean availability (proportion of time spent on surface) was 0.633 (SD = 0.094) for ‘on shelf’, and 0.136 (SD = 0.075) for ‘off shelf’ with this Bayesian model. These predicted values were used in the abundance models to correct for availability bias.

**Table 1:** Posterior inferences of the coefficients (on the logit-scale) in the best Bayesian zero-one-inflated beta distribution model on blue sharks’ proportion of time spent at the surface (0–1 m). The first model component estimates the mean (linear predictor) in the model, and the second and third component the probability of zero and one, respectively. The factor levels ‘off shelf’ and ‘time bin’ 2 (06:00–12:00) are the baseline values in the model and are included in the intercept. Time bin 3 is the time period 12:00–18:00.

Model component	Effect	Estimate	SE	2.5% quantile	97.5% quantile
logit(mean)	Intercept	-1.110	0.005	-1.570	-0.686*
	as.factor(Shelf)—on	1.889	0.006	1.386	2.407*
	as.factor(Time bin) - 3	-0.236	0.003	-0.464	-0.013*
logit(Pr(y = 0))	Intercept	-0.364	0.007	-0.939	0.208
	as.factor(Shelf)—on	-5.757	0.031	-8.799	-3.828*
	as.factor(Time bin) - 3	0.643	0.009	-0.118	1.419
logit(Pr(y = 1))	Intercept	-71.576	0.863	-160.022	-17.622*
	as.factor(Shelf)—on	34.001	0.662	-0.438	102.495
	as.factor(Time bin) - 3	32.529	0.648	0.714	103.489*
	d	1.828	0.004	1.509	2.120*
	σ	1.273	0.006	0.817	1.815*

d—Regression coefficient in the linear predictor for the sum of the two shape parameters in the beta distribution

σ—Posterior mean of the variance of the random effect

* indicates a significant difference when the quantile range does not overlap zero

**Fig 2:**
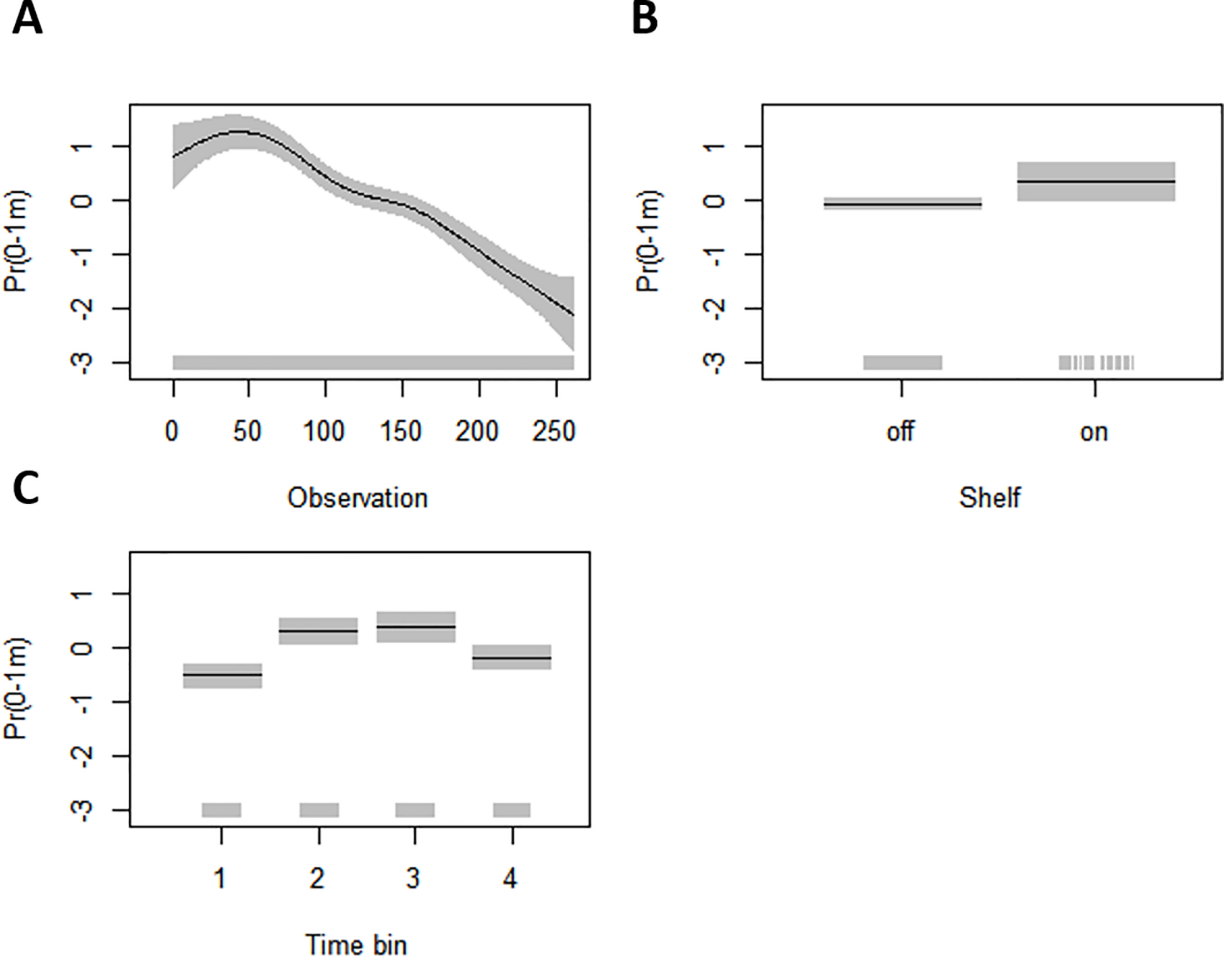
Partial residual plots (on the logit-link scale) of the significant co-variates in the best GAMLSS model for time spent at the surface for shark A. Estimated relationship between the response and (A) time (observation), (B) position on or off continental shelf, and (C) time bin (1 = 00:00–06:00, 2 = 06:00–12:00, 3 = 12:00–18:00, 4 = 18:00–24:00). The black line is the mean, the grey bars represent the standard error, and the rug plot on the x-axis shows the actual data values.

**Fig 3:**
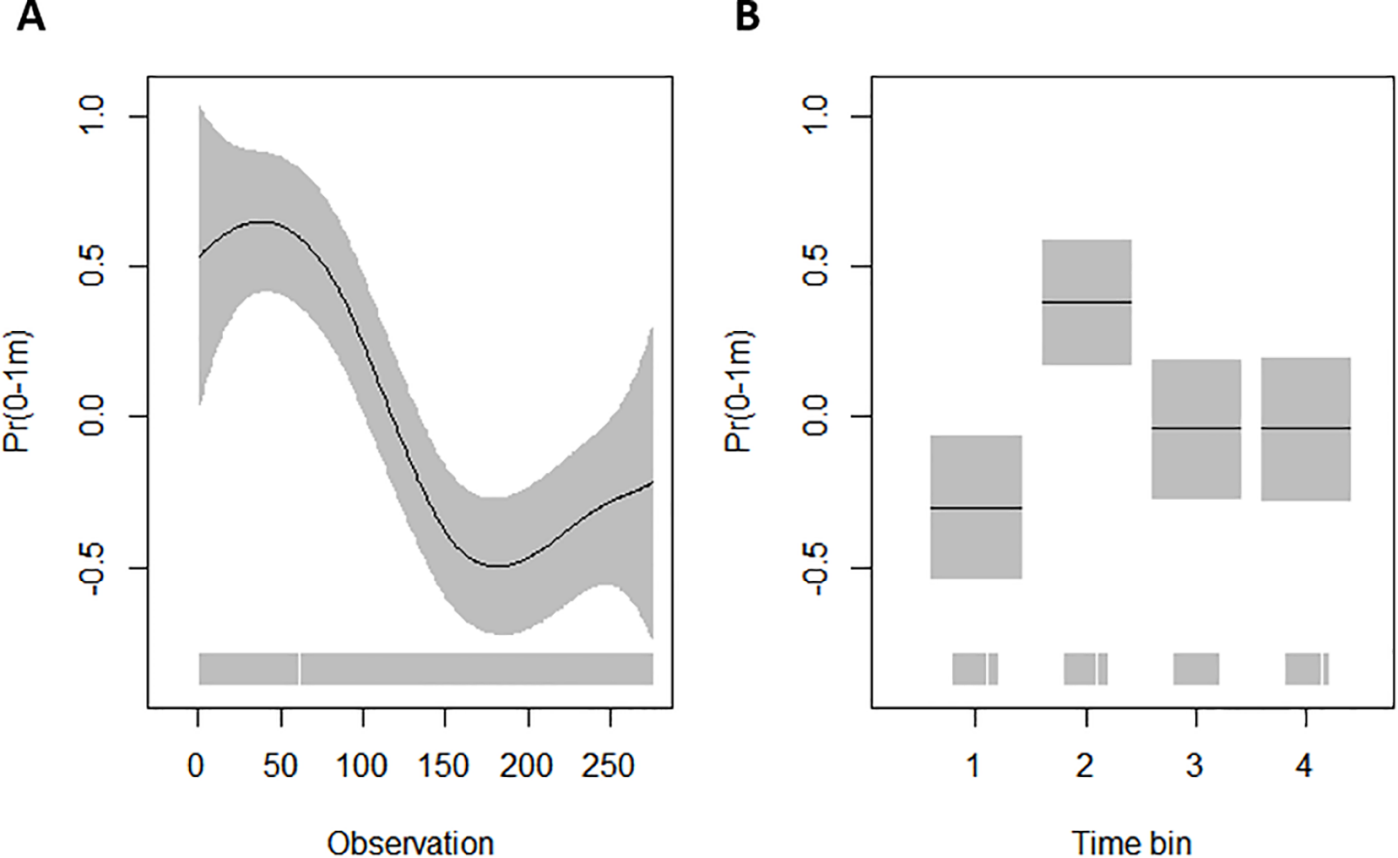
Partial residual plots (on the logit-link scale) of the significant co-variates in the best GAMLSS model for time spent at the surface for shark B. Estimated relationship between the response and (A) time (observation), and (B) time bin (1 = 00:00–06:00, 2 = 06:00–12:00, 3 = 12:00–18:00, 4 = 18:00–24:00). The black line is the mean, the grey bars represent the standard error, and the rug plot on the x-axis shows the actual data values.

From the GAMLSS models run separately for each shark, the best model explaining the time spent at the surface for shark A included a cubic smooth of time (running observation), as well as factor variables ‘time bin’ and ‘shelf’ (AIC: -56.14). The next best model included the same terms and also an interaction between ‘shelf’ and ‘time bin’ (AIC: -52.95). All the terms in the best model for shark A were significant (Table 2) and the relationship of the response with each term is presented in Fig 2. Shark A spent proportionally more time at the surface during the 50–60 first observations (~15 days) after which it started to spend more time at deeper depths (Fig 2A). Proportion of time spent at the surface was significantly higher whilst on shelf (Table 2, Fig 2B), and during daytime, i.e. between the hours of 06:00 and 18:00 (Table 2, Fig 2C). Shark B never made dives to depth >200 m and it was assumed, based on the recreated path (S2 Fig), that it never left the shelf waters. Therefore, the time at the surface was modelled only using two co-variates, observation and time bin. The best GAMLSS-model included both of these terms (AIC: -31.81) and their effect on the response are presented in Table 3 and Fig 3. Similar to shark A, shark B spent proportionally the most time in the surface waters during the first 50–60 observations after which it started spending more time in deeper depths (Fig 3A). shark B also spent significantly less time at the surface during night time (00:00–06:00) and most time at the surface during the morning hours (06:00–12:00) (Fig 3B).

**Table 2:** Coefficients, their standard errors (SE), t-values and significance of the terms in the best GAMLSS-model explaining the mean proportion of time spent at 0-1m by shark A. The factor levels ‘off shelf’ and ‘time bin’ 1 (00:00–06:00) are the baseline values in the model and are included in the intercept. Time bin 2 = 06:00–12:00, 3 = 12:00–18:00 and 4 = 18:00–24:00.

Model terms	Estimate	SE	t-value	Pr(>|t|)
Intercept	-0.7120	0.2344	-3.0380	0.0026
cs(observation)	-0.0125	0.0014	-9.1770	<0.001
as.factor(Shelf)—on	0.4102	0.2179	1.8830	0.0609
as.factor(Time bin) - 2	0.8226	0.1789	4.5990	<0.001
as.factor(Time bin) - 3	0.9025	0.2011	4.4880	<0.001
as.factor(Time bin) - 4	0.3444	0.1658	2.0770	0.0388

**Table 3:** Coefficients, their standard errors (SE), t-values and significance of the terms in the best GAMLSS-model explaining the mean proportion of time spent at 0-1m by shark B. The factor level ‘time bin’ 1 (00:00–06:00) is the baseline value and included in the intercept. Time bin 2 = 06:00–12:00, 3 = 12:00–18:00 and 4 = 18:00–24:00.

Model terms	Estimate	SE	t-value	Pr(>|t|)
Intercept	0.9659	0.1922	5.0260	<0.001
cs(observation)	-0.0049	0.0009	-5.3110	<0.001
as.factor(Time bin) - 2	0.6792	0.1856	3.6600	<0.001
as.factor(Time bin) - 3	0.2605	0.1988	1.3110	0.1911
as.factor(Time bin) - 4	0.2592	0.1997	1.2980	0.1955

### Abundance estimation

**Fig 4:**
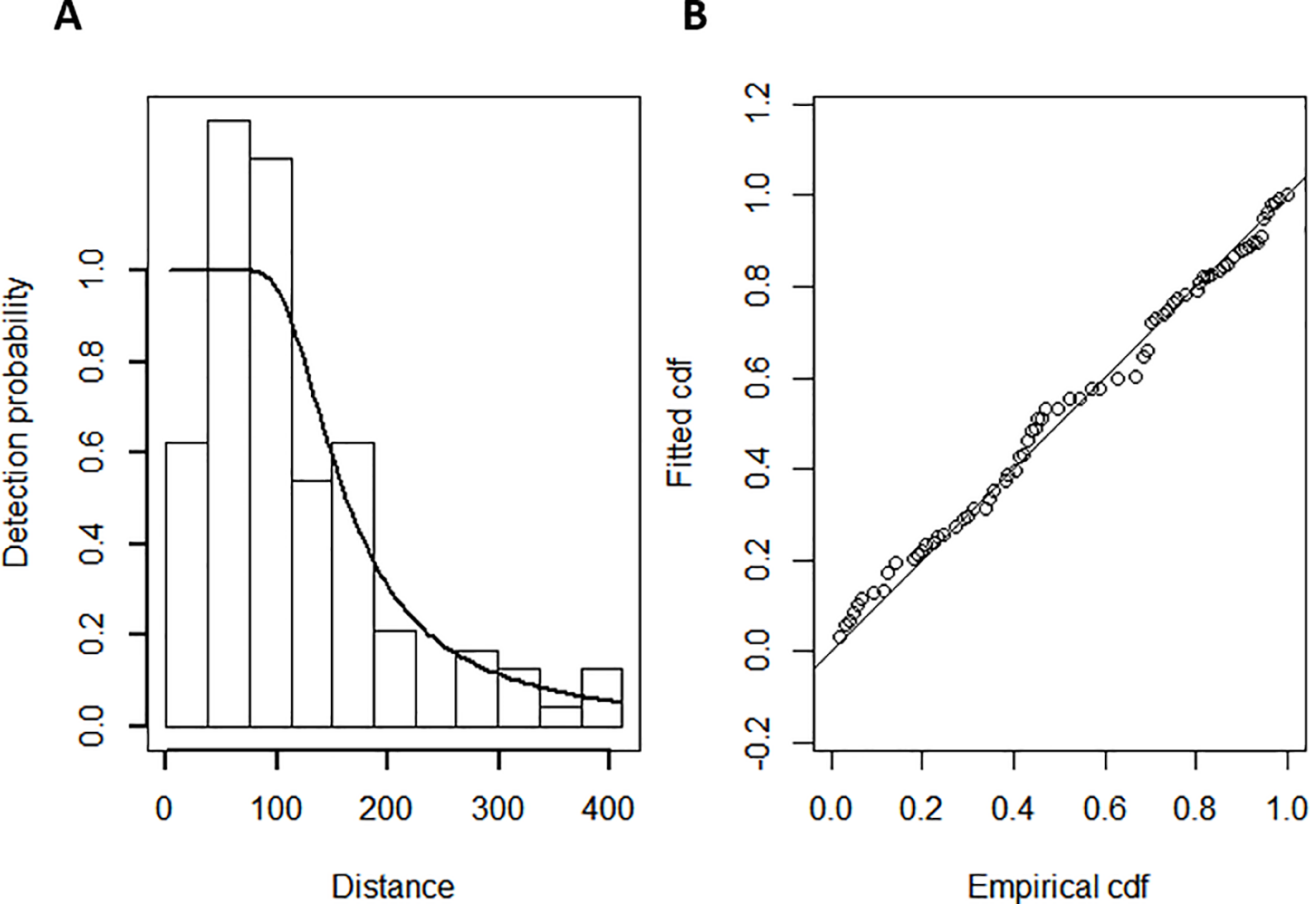
(A) Detection probability of sharks as a function of distance, and (B) the goodness-of-fit of the best detection function, a Hazard-rate model including an index of cloud cover.

The detection function, including an index of cloud cover, was determined as the best model to explain the variation in the detection probability of sharks (Table 4, Fig 4). While the AIC value for this model was the second lowest, this model was chosen based on its better fit to the data (Cramer-von-Mises *p* = 0.544) compared to the model with the lowest AIC (Cramer-von-Mises *p* = 0.098).

**Table 4:** Models tested to estimate the detection function as part of distance sampling abundance estimation of blue sharks in the Irish Exclusive Economic Zone.

Model type	Co-variates	AIC	Cramer-von-Mises *P*
Hazard rate	sea state, subjective conditions, glare intensity, cloud cover	1365.261	0.098
Hazard rate	cloud cover	1369.405	0.544
Hazard rate	sea state, cloud cover	1371.205	0.648
Hazard rate	subjective conditions, cloud cover	1371.608	0.511
Hazard rate	null	1372.248	0.389
Hazard rate	subjective conditions, glare intensity, cloud cover	1372.440	0.395
Hazard rate	sea state, subjective conditions, cloud cover	1372.790	0.814
Hazard rate	glare intensity, cloud cover	1373.128	0.811
Hazard rate	sea state	1373.279	0.625
Hazard rate	sea state, glare intensity, cloud cover	1374.477	0.815
Hazard rate	subjective conditions	1374.851	0.357
Hazard rate	glare intensity	1377.135	0.587
Half-normal	null	1378.953	0.196
Hazard rate	turbidity	Failed to fit	Failed to fit

**Fig 5:**
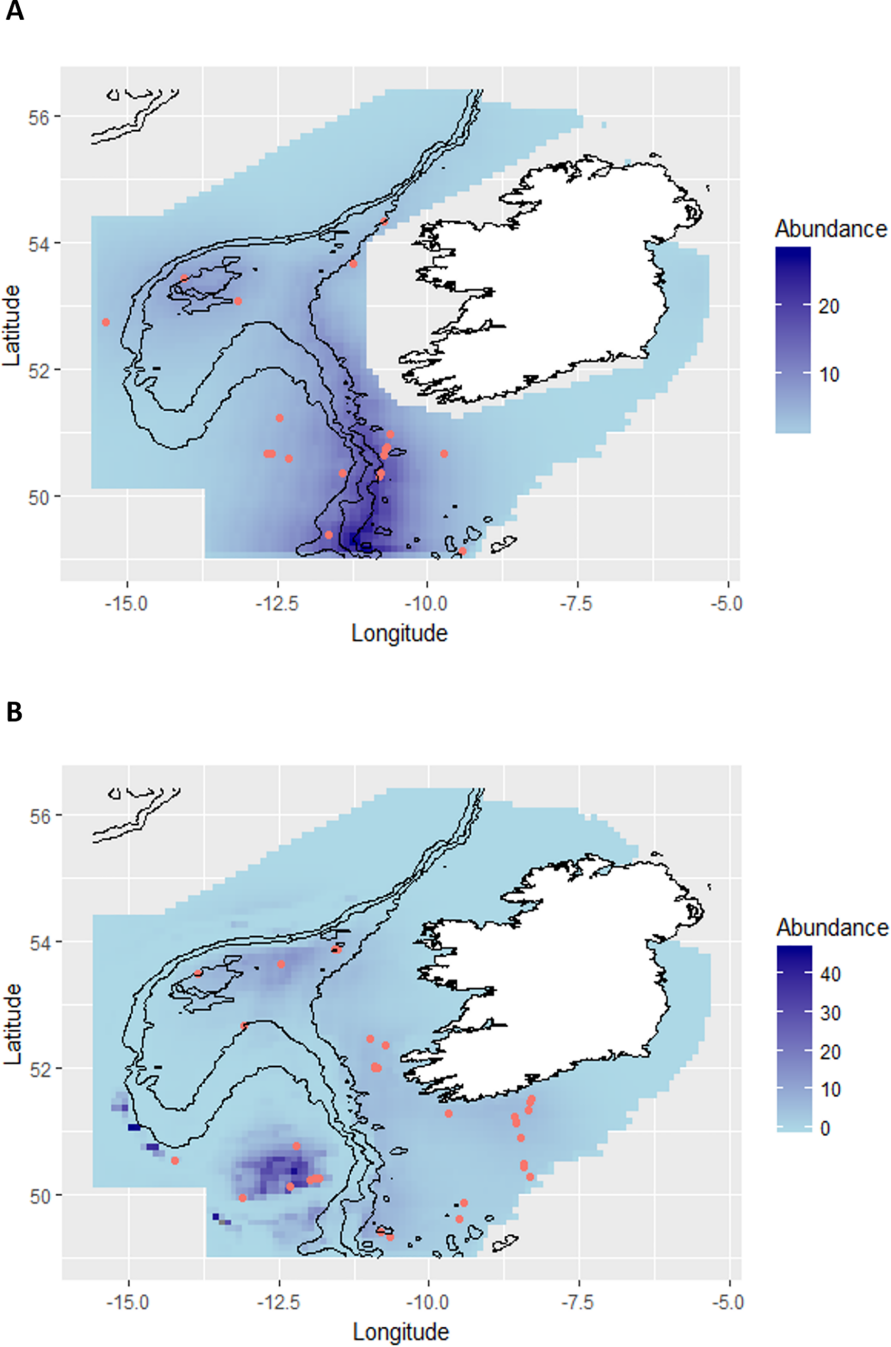
Predicted abundance for blue sharks in the survey area (A) in summer 2015, and (B) in summer 2016, corrected with modelled availability bias. Red circles denote blue shark sighting locations. The contours shown are 200m, 500m, and 1000m depth contours.

In 2015, the best model for the abundance of blue sharks included the two-dimensional spatial smooth and the covariate ‘distance to 200 m contour’, with and without accounting for availability bias. These two co-variates explained little over a quarter of the variation in blue shark abundance with a deviance of 26.3% in all three models. In 2015, the predicted mean abundance of blue sharks in the whole study area was 2,218 (CV = 0.24, 95% CI: 1,394–3,532) without correcting for availability bias, 5,412 (CV = 0.24, 95% CI: 3,400–8,616) with average bias correction, and 15,320 (CV = 0.28, 95% CI: 9,011–26,046) with the model-predicted bias correction (Fig 5A).

In 2016, the best uncorrected model and the model corrected with average availability included the spatial component and the co-variates ‘slope index’, ‘distance to shore’ and ‘Chl-a’, with these terms explaining 34.9% of the variation in blue shark abundance. The predicted uncorrected abundance throughout the study area was 3,577 (CV = 0.21, 95% CI: 2,374–5,389), and with the model corrected with average availability it was 8,724 (CV = 0.21, 95% CI: 5,790–13,144). With model-predicted availability bias, the best abundance model included the spatial smooth and the terms ‘slope index’ and ‘distance to shore’, with 43.5% deviance explained. With this model, the predicted mean blue shark abundance for the whole study area was 11,001 (CV = 0.27, 95% CI: 6,553–18,471) (Fig 5B).

## Discussion

There is worldwide concern about the status of elasmobranchs, primarily as a result of overfishing and bycatch [7,9,12] with subsequent ecosystem effects following the removal of top predators [41,42]. Rapid declines of blue sharks of 50–80% over a few generations have been reported in parts of the Atlantic [12], and it is estimated that over 10 million blue sharks are killed annually [43]. In the area surveyed for this study alone, catches from recreational fisheries have undergone a marked decline since the early to mid-1990s [15], highlighting the need for robust abundance estimates of this species. In this study, the absolute abundance of blue sharks, accounting for bias in the observation process as well as variation in availability bias resulting from the animals spending time at depth, was estimated for the first time. Moreover, this abundance estimate covers a large area extending over ca. 427,000 km^2^ of the Irish Exclusive Economic Zone. This area consists of varying marine habitats that form a significant part of the Celtic-Biscay Shelf Large Marine Ecosystem in the Northeast Atlantic. Availability for detection was modelled as the proportion of time that blue sharks spent within the top meter of the water column, and it was found that time of day and sharks’ position on or off the continental shelf had a significant effect with more time spent at the surface at daytime and whilst over the shelf. Depending on the year, the abundance estimate corrected with modelled availability bias was up to seven times higher compared to the estimate without the bias correction, and nearly three times higher compared to the abundance corrected with average availability.

Both of the tagged blue sharks spent significantly less time at the top layer of the water column between midnight and 6am, with the largest proportion of time spent at the surface in the morning and afternoon (6 am– 6 pm) by shark A and in the morning (6 am– 12 am) by shark B (Figs 2C and 3B). Other studies have recorded similar behaviour with blue sharks being confined to depths near the thermocline at night whilst making repeated vertical round-trips between the surface and depths of hundreds of meters during daytime [26]. Doyle et al. [24] found an increase in “knifing” behaviour, where blue sharks were swimming at the surface with their dorsal fins breaking the water, at post-dawn, and hypothesized that blue sharks are taking advantage of the changing light conditions to surprise prey silhouetted at the surface [24]. Preference for surface waters during daylight hours has also been reported with blue sharks [25] and basking sharks [44] tagged in the English Channel, and this has been linked to reverse diel vertical migration of zooplankton in the inner-shelf areas of the Northeast Atlantic that are characterized by tidal fronts and high productivity [45] attracting large schools of pelagic fish [45]. Even though the diet of blue sharks caught off-shore consists mainly of pelagic squid [43,46], they are known to feed on pelagic fish such as clupeids and mackerel (*Scomber scombrus*) in the English Channel [47]. Another possible explanation for the preference for surface waters during daylight hours could be related to physiology and thermal recovery after deep diving [48,49]; however, Doyle et al. [24], who used the same PSAT data used in this study, found very little evidence for thermal recovery as the mean temperature experienced by a blue shark during night versus day differed only by <0.2°C [24].

There was a strong on/off-shelf signal in the data, with a model-predicted average of 63% of time spent at the surface in shelf waters, reducing to 14% of time at the surface in off-shelf waters. Queiroz et al. [2] found a similar shift in depth distribution between on- and off-shelf waters in southwest England, although the on-shelf versus off-shelf depth use of blue sharks varied between individuals and years [2]. The most likely path reconstructed for shark B, coupled with the lack of dives to depths of >200 m, indicated that this juvenile shark remained in shelf waters throughout the deployment period, consistent with Quieroz et al. [2] who also found that juvenile sharks generally preferred to stay within productive shallow shelf waters [2].

Distance to the continental shelf (200 m contour) was a significant factor explaining the variance in the abundance of blue sharks in both years of the study, with larger numbers predicted in areas around the shelf margin and around the shallow areas on the Porcupine Bank, especially in 2015 (Fig 5A). Continental shelf break is generally considered as a location where tidal fronts form (e.g. [50,51]), and the margins of the Northeast Atlantic shelf are productive areas with upwelling and mixing [52] acting as spawning grounds for numerous fish species [53], which might in turn attract blue sharks and other top predators to these areas. In 2016, chlorophyll-a concentration was found to be a significant predictor of blue shark abundance, with the predicted blue shark high density area concentrated on small areas along the southern continental shelf margin and especially around the Porcupine Seabight (Fig 5B). This region is thought to support rich biodiversity with aggregations of other megafauna, such as large baleen whales, reported in the area [54].

Despite the extensive survey effort in the winter (equal to summer), the lack of sightings of blue sharks during the winter surveys suggests that the species does not occur, at least in large numbers, in the survey area over the winter months. This is consistent with the reported cyclical seasonal migrations to warmer lower-latitude waters during the winter across the Atlantic (e.g. [3,4,55,56]), and the observed southerly migration of five blue sharks satellite-tagged in Irish waters [24]. The modelled availability-corrected abundance estimates for both summers (2015 and 2016) were similar in scale (15,300 and 11,000, respectively) with largely overlapping confidence intervals, despite the fact that the abundance was predicted for a smaller area in 2015. The surveys in different years were timed to take place at the same time of the year, and the similarity in the estimates may indicate no large-scale change in the population between the two years; however, the power to detect a change between the two years is likely to be lacking in this study thus preventing robust comparison between the yearly estimates. Nevertheless, relative abundance estimates based on CPUE in Irish waters show no increase or decrease in catches since the early 2000s, after the apparent decline in CPUE in the mid-1990s [15]. The effect of including a model-predicted versus average availability bias in the abundance estimation varied between years, with a smaller difference (26%) between the two estimates in 2016, compared to the 2015 bias-corrected estimates, which differed by 280%. This is likely an artefact of a comparatively larger number of sightings occurring in shelf waters during the second summer of surveys with the extended survey area.

The mean cholorophyll-a concentration across the study area was similar between the years and months of the tagging and the summer aerial surveys, with variation around the mean decreasing during winter months (see S1 Fig). This makes it unlikely that the differences in the monthly chlorophyll-a have affected the diving patterns of blue sharks and their time spent at the surface. The mean monthly temperature across the study area varied from ~10°C– 15.5°C during the tagging months, whereas it remained between ~12°C and 15°C during the summer aerial surveys, and these changes in temperature may have affected the diving behaviour of blue sharks. Vianna and colleagues [1], for example, found grey reef sharks (*Carcharhinus amblyrhynchos*) to prefer deeper depths in the spring following an increase in water temperature at these depths. However, the reconstructed track of one of the tagged blue sharks (shark B; see S2 Fig) showed this shark moving southwards and likely into warmer waters so it is probable that the temperature range over the tagging period experienced by the sharks was even smaller than that presented in S1 Fig.

Aerial surveys have become widely used in cetacean abundance estimation, especially over large or remote areas, and they have recently been applied to other marine taxa ranging from sharks [20–22] and rays [23] to bony fish [57,58] and turtles [59]. Furthermore, dedicated aerial surveys for marine mammals provide platforms of opportunity for recording and estimating the abundance of vulnerable or data-poor marine species. This study demonstrates the importance of including availability bias in the abundance estimation of species that spend time at depth. Moreover, we show that availability bias can vary both spatially and temporally, albeit our predictions are based on a small sample size assumed to represent typical behaviour of blue sharks in the area. Dive data from a larger number of animals, representing both sexes and different maturities, and collected at the same time and across the range of the aerial surveys to potentially minimize variation in the environmental conditions, would likely increase the precision and power of the abundance estimate. Nevertheless, this study serves as an example of a methodology that can be applied in future studies when correcting for availability bias in abundance estimation for various diving taxa. In particular, if the survey area includes a wide variety of environments ranging from shallow to deep waters, modelling availability provides a more robust alternative than applying average time at the surface estimates, producing more realistic abundance estimates necessary for effective management and conservation of species. Modelling availability bias has wider applications in abundance studies of other taxa such as marine turtles, whose surfacing patterns may vary due to environmental factors such as temperature, bathymetry [60] or changes in vertical distribution of prey [61].

## Supporting information

S1 Fig Sea Surface Temperature (SST) and Chlorophyll-a concentration (Chl-a) across the area where the aerial surveys were conducted, over the tagging period and during the summer aerial surveys. Mean is denoted with black circles and the whiskers represent standard deviation. The data for the plots were retrieved from https://oceancolor.gsfc.nasa.gov/cgi/l3.
(JPG)

S2 Fig Most likely swimming path (red circles) of shark B reconstructed from pop-up satellite archival tag (PSAT) light intensity data. The first contour line from land depicts 150m depth contour The map was created using the R-package ‘marmap’ [62].
(PNG)

S3 Fig Observed (x-axis) and predicted (y-axis) blue sharks’ mean proportion of time at the surface (0-1m) with the best zero-one inflated beta distribution model using Bayesian inference. This model included a random intercept comprised of Shark ID and day and factor variables ‘shelf’ and ‘time bin’. The solid line represents a perfect fit of the model to the data.
(PNG)

S1 Table Start and end days and effort during aerial surveys of the Irish Exclusive Economic Zone. (DOCX)

S2 Table Co-variates used in the density surface modelling of abundance of blue sharks. (DOCX)

S3 Table Blue shark availability data. (XLSX)

S4 Table Data used in density surface models for blue shark abundance. (XLSX)
