# Supplementary figures and images for: Using tagging data and aerial surveys to incorporate availability bias in the abundance estimation of blue sharks (Prionace glauca)
Source: PLoS One. 2018 Sep 11;13(9):e0203122. doi: 10.1371/journal.pone.0203122 (PMC6133345; doi:10.1371/journal.pone.0203122)

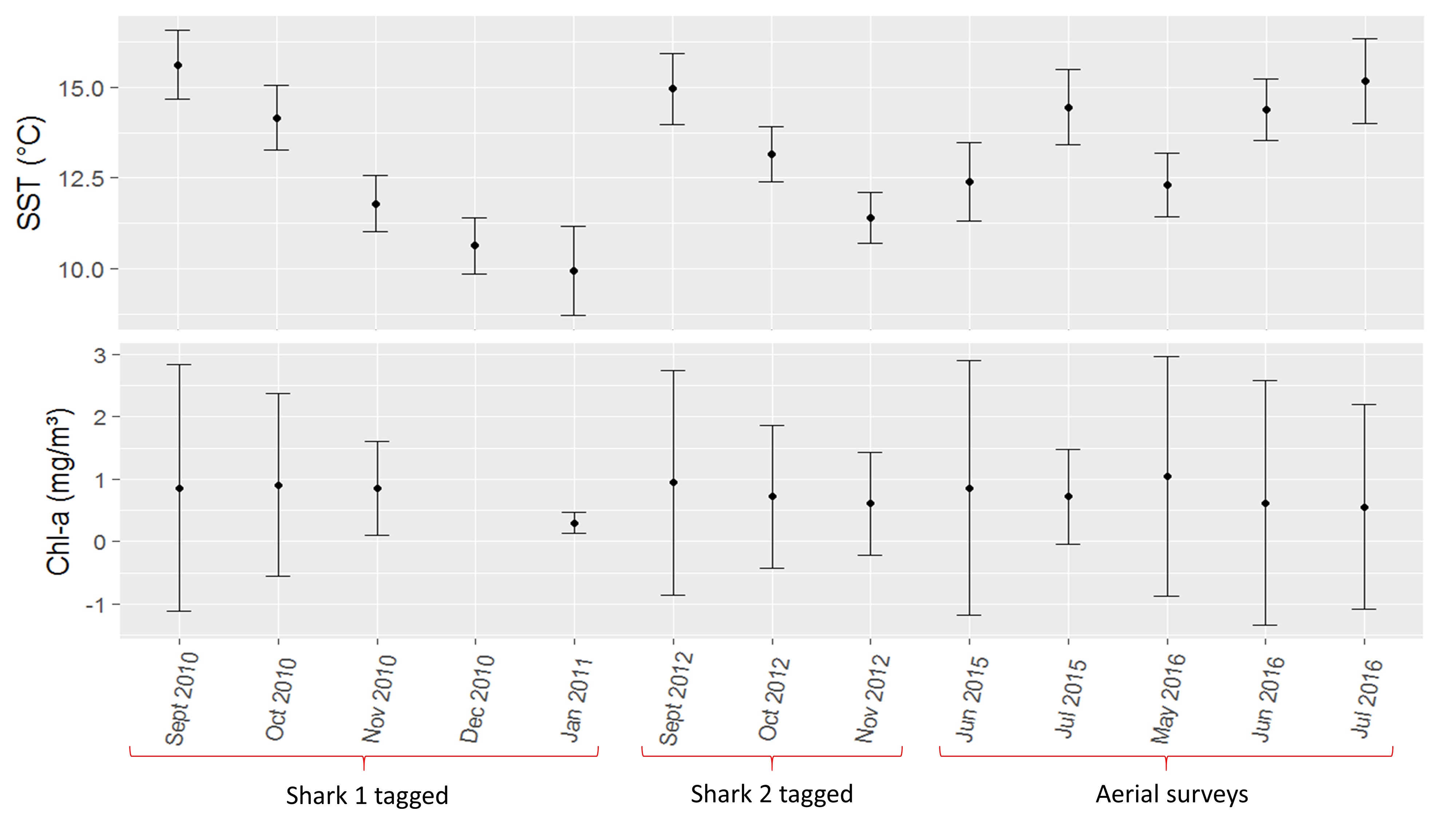

Supplement: S1 Fig — Mean is denoted with black circles and the whiskers represent standard deviation. The data for the plots were retrieved from https://oceancolor.gsfc.nasa.gov/cgi/l3. (JPG) [file pone.0203122.s001.jpg]

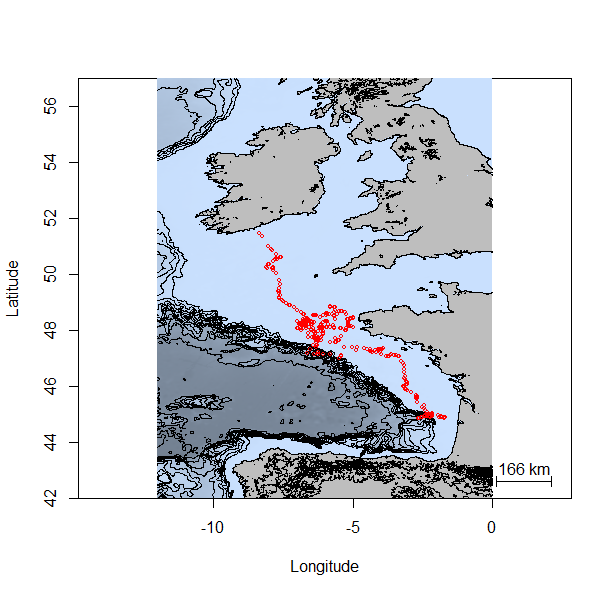

Supplement: S2 Fig — The first contour line from land depicts 150m depth contour The map was created using the R-package ‘marmap’ [62]. (PNG) [file pone.0203122.s002.png]

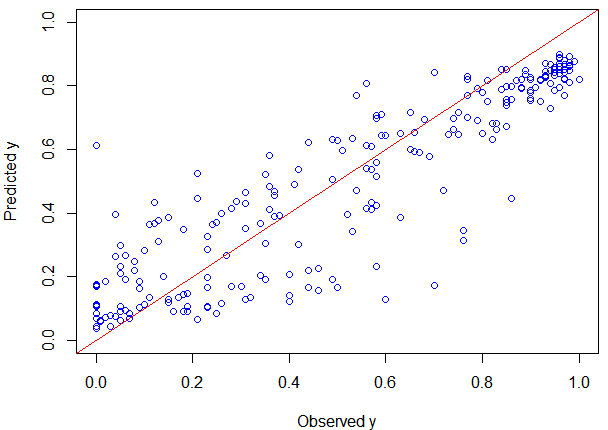

Supplement: S3 Fig — This model included a random intercept comprised of Shark ID and day and factor variables ‘shelf’ and ‘time bin’. The solid line represents a perfect fit of the model to the data. (PNG) [file pone.0203122.s003.png]
