# Supplementary material for: Using tagging data and aerial surveys to incorporate availability bias in the abundance estimation of blue sharks (Prionace glauca)
Source: PLoS One. 2018 Sep 11;13(9):e0203122. doi: 10.1371/journal.pone.0203122 (PMC6133345; doi:10.1371/journal.pone.0203122)
Supplement: S1 Table — (DOCX) [file pone.0203122.s004.docx]

**S1 Table** Start and end days and effort during aerial surveys of the Irish Exclusive Economic Zone.

| **Survey** | **Start date** | **End date** | **Effort (km)** |
| --- | --- | --- | --- |
| Summer 2015 | 08/06/2015 | 15/07/2015 | 8,027 |
| Winter 2015-16 | 03/11/2015 | 28/02/2016 | 8,770 |
| Summer 2016 | 21/05/2016 | 07/07/2016 | 10,244 |
| Winter 2016-17 | 02/11/2016 | 15/03/2017 | 10,143 |
