# Supplementary material for: Using tagging data and aerial surveys to incorporate availability bias in the abundance estimation of blue sharks (Prionace glauca)
Source: PLoS One. 2018 Sep 11;13(9):e0203122. doi: 10.1371/journal.pone.0203122 (PMC6133345; doi:10.1371/journal.pone.0203122)
Supplement: S2 Table — (DOCX) [file pone.0203122.s005.docx]

**S2 Table** Co-variates used in the density surface modelling of abundance of blue sharks.

| **Name** | **Description** | **Source** |
| --- | --- | --- |
| **Depth** | Average depth in the grid cell | 2-Minute Gridded Global Relief Data (ETOPO2v2). National Geophysical Data Center (NGDC). NOAA Satellite and Information Service. |
| **Distance to land** | Distance to the 0 m depth contour (coast), in decimal degrees | Calculated with the Spatial Analyst extension of ArcGis 9.2, using GEBCO bathymetric data. |
| **Distance to 200m contour** | Distance to the 200 m depth contour, in decimal degrees | Calculated with the Spatial Analyst extension of ArcGis 9.2, using GEBCO bathymetric data. |
| **Slope index** | Slope of the sea floor in m per km, calculated as follows: $\frac{\left(\frac{depth_{\max}-depth_{\min}}{dis\tan ce in km_{(depth_{\max}-depth_{\min})}}\right)}{10}$  | Derived from ETOPO2 bathymetric data |
| **SST** | Average Sea Surface Temperature for the months of May to August 2015 and 2016. | Sensor: Moderate Resolution Imaging Spectroradiometer (MODIS) on Aqua, Advanced Very High Resolution Radiometer (AVHRR) on POES, Imager on GOES, Advanced Microwave Scanning Radiometer (AMSR-E) on Aqua. Resolution: 0.1 degrees. NOAA CoastWatch Program |
| **Chlorophyll-a** |  | Chlorophyll *a* (mg/m³) was available from the NEO NASA website as a floating point GeoTIFF. Resolution 0.1 degrees. |
| **Mixed layer depth (MLD)** | Monthly modelled means of 15^th^ of each month. | Downloaded from the Marine Institute Data Portal (<http://data.marine.ie/>). Resolution was available as 0.025dd grid squares and scaled up to 0.1dd |
